# The SOFIA pilot study: assessing feasibility and fidelity of coordinated care to reduce excess mortality and increase quality of life in patients with severe mental illness in a general practice setting; a cluster-randomised pilot trial

**DOI:** 10.1186/s12875-023-02141-2

**Published:** 2023-09-16

**Authors:** Katrine Tranberg, Alexandra Jønsson, Tina Due, Volkert Siersma, John Brandt Brodersen, Kristine Bissenbakker, Frederik Martiny, Annette Davidsen, Pia Kürstein Kjellberg, Kevin Doherty, Stewart W. Mercer, Maria Haahr Nielsen, Susanne Reventlow, Anne Møller, Maarten Rozing, on behalf of the SOFIA Study Group

**Affiliations:** 1 Department of Public Health, The Section of General Practice and the Research Unit for General Practice, University of Copenhagen, Copenhagen, Denmark; 2 Department of People and Technology, Roskilde University, Roskilde, Denmark; 3 Department of Community Medicine, Faculty of Health Sciences, The Research Unit for General Practice, UiT The Arctic University of Norway, Tromsø, Norway; 4 The Mental health services in the Capital Region of Denmark, Copenhagen, Denmark; 5 The Primary Health Care Research Unit, Region Zealand, Zealand, Denmark; 6 Department of Social Medicine, Bispebjerg and Frederiksberg Hospital, Copenhagen, Denmark; 7 VIVE - The Danish Center for Social Science Research, Copenhagen, Denmark; 8 Copenhagen Center for Health Technology (CACHET), Department of Health Technology, Technical University of Denmark, Lyngby, Denmark; 9 Old Medical School, Usher Institute, University of Edinburgh, Edinburgh, UK; 10 Psychiatric Center Copenhagen, Copenhagen, Denmark

**Keywords:** Complex intervention, General practice, Extended consultations, Pilot studies, Severe mental illness, Recruitment strategies, Coordinated care program

## Abstract

**Objective:**

To evaluate the feasibility and fidelity of implementing and assessing the SOFIA coordinated care program aimed at lowering mortality and increasing quality of life in patients with severe mental illness by improving somatic health care in general practice.

**Design:**

A cluster-randomised, non-blinded controlled pilot trial.

**Setting:**

General Practice in Denmark.

**Intervention:**

The SOFIA coordinated care program comprised extended structured consultations carried out by the GP, group-based training of GPs and staff, and a handbook with information on signposting patients to relevant municipal, health, and social initiatives.

**Patients:**

Persons aged 18 years or older with a diagnosis of psychotic, bipolar, or severe depressive disorder.

**Main outcome measures:**

We collected quantitative data on the delivery, recruitment and retention rates of practices and patients, and response rates of questionnaires MMQ and EQ-5D-5 L.

**Results:**

From November 2020 to March 2021, nine practices were enrolled and assigned in a 2:1 ratio to the intervention group (n = 6) or control group (n = 3). Intervention group practices included 64 patients and Control practices included 23. The extended consultations were delivered with a high level of fidelity in the general practices; however, thresholds for collecting outcome measures, and recruitment of practices and patients were not reached.

**Conclusion:**

Our findings suggest that delivering the coordinated care program in a fully powered trial in primary care is likely feasible. However, the recruitment methodology requires improvement to ensure sufficient recruitment and minimize selective inclusion.

**Trial registration:**

The date of pilot trial protocol registration was 05/11/2020, and the registration number is NCT04618250.

## Background

People with a severe mental illness (SMI), defined as schizophrenia, other primary psychotic disorders, and severe affective disorders, have a marked reduction in life expectancy [1–3] and the mortality gap between people with and without SMI has increased during the past decades [4]. The majority of deaths in people with SMI are due to chronic somatic diseases, i.e. cardiovascular diseases, cancer, and respiratory diseases [5]. The underlying mechanisms are multifaceted, including high exposure to metabolic risk factors, adverse effects of psychotropic medication, genetic predisposition, and inequality and inequity in the provision and utilization of health care [6]. Alleviating the barriers for people with SMI in general practice and thereby promoting access to screening, preventive interventions, and improved somatic health care, may ultimately lead to a reduction in excess mortality and improve health-related quality of life. However, there is little evidence about the feasibility and effectiveness of interventions aiming to alleviate barriers to somatic health care in general practice for patients with SMI [7].

During a 2-year participatory co-design process, we developed the SOFIA coordinated care program that attempts to align the health care provision in primary care with the needs and preferences of people with SMI [8]. The program comprised education of general practitioners (GPs) and staff, a handbook providing an overview of relevant signposting and referral options, and an extended consultation for patients with SMI with their GP following a structured consultation model, termed the SOFIA scheme (SOcial, FInding, and Agreement) [9]. Previously, we tested the feasibility and acceptability of introducing extended consultations and the clinical content in a general practice setting in a small-scale non-randomized intervention-arm-only feasibility trial [10]. It was perceived as acceptable to both GPs and patients with SMI [10]. However, as uncertainties remained concerning the intervention’s educational component, optimal procedures for randomisation, patient recruitment and retention, fidelity and intervention delivery, and data collection, we conducted a cluster-randomized pilot trial in preparation for a large-scale RCT.

To clarify the terminology used in this work, we refer to the intervention and its components as the ‘SOFIA program’ or ‘the coordinated care program’ and the research activities performed to develop, evaluate, and assess the program (e.g., the feasibility and pilot study) as ‘the SOFIA study’. The SOFIA program aims to lower mortality and improve the quality of life in patients with SMI. The SOFIA study aims to evaluate whether the coordinated care program is successful in reducing excess mortality and increasing the quality of life in patients with severe mental illness, as well as assessing the implementation in the context of general practice. The goal of the SOFIA pilot study is to investigate the implementation and feasibility of introducing the coordinated care program into the context of general practice which will be addressed in a series of publications. The current paper aims to address:

1. The feasibility of the design in terms of recruitment of practices and patients, and the retention of practices and patients.
2. The fidelity with which GPs delivered the extended consultation.
3. The feasibility of collecting outcome measures (response rates on MMQ and EQ-5D-5L questionnaires); and thereby obtaining preliminary data to inform the required sample size for a subsequent, large-scale efficacy trial.
4. Information on selected aspects of the program theory relating to the extended consultations and the educational component.

Questions assessing the benefits and potential disadvantages regarding the chosen recruitment strategy and the use of the MMQ questionnaire as an intervention tool and outcome measure will be elaborated in upcoming publications.

## Materials and methods

### Settings and study design

The pilot study methodology has been reported previously in a protocol article [9] and is briefly described below. The study was designed as a cluster-randomised, non-blinded, parallel-group trial including general practices from the Capital Region of Denmark (excluding the island of Bornholm) and Region Zealand. Patient recruitment started on November 6th, 2020. The study was completed on September 30th, 2021.

### Participating practices

General practices were invited to participate by the research team and were contacted by phone and email during opening hours. We aimed to recruit six general practices from the Capital Region and six general practices from Region Zealand.

### Patient inclusion criteria

Patients were eligible to participate if they were aged 18 and older and were diagnosed with an SMI prior to study enrolment. SMI was defined as; (A) *psychotic disorder*; (B) *bipolar disorder*; or (C) a *severe depressive disorder*.

### Identification, eligibility assessment, and recruitment of patients with SMI

General practices collected data on patient diagnoses and medication prescriptions from their electronic medical records. A computer algorithm selected a random sample of 45 patients who met the inclusion criteria in each practice. This selection was stratified, and the lists were returned to the GP. Each sample of 45 patients contained three groups of 15 patients who fulfilled diagnostic criteria A, B, or C respectively. The GP was instructed to verify the diagnosis and assess patient eligibility. GPs were instructed to recruit a minimum of two patients from each diagnostic subgroup A, B, and C. A reimbursement limit of 15 patients per practice was used. Reimbursement was provided by the SOFIA study and is not a part of the custom Danish monetary compensation. GPs or practice staff contacted potentially eligible patients and obtained written informed consent.

### Exclusion criteria

Patients were excluded if they were subjected to any type of legal measure as stipulated in the Danish Mental Health Care Act, registered with a dementia diagnosis, organic psycho-syndrome or other neurological diseases; were receiving end-of-life care; were non-Danish speakers; or if the psychiatric diagnosis appeared to be incorrect or outdated. GPs were instructed to inform the research team if they evaluated the patient’s overall functional level as too low for meaningful participation in the trial. In such instances, the research team decided on in- or exclusion in dialogue with the GP.

### The coordinated care program

Besides the overall goal, the SOFIA program seeks to strengthen the patient’s perspective in health care decisions. The coordinated care program consists of four key components: (1) GPs in the intervention group attended a mandatory one-day introductory course. Due to COVID-19 restrictions, the planned two-day course for GPs was shortened to a single-day course. (2) GPs received a specialised handbook, specific to their municipal and regional affiliation, which contained the contact information of relevant social and health care actors, and helplines in case of substance abuse or risk of suicide or self-harm. The handbook provided information on relevant signposting possibilities for the patient group. (3) Patients were invited to an extended consultation by their GP following the SOFIA scheme [9]. (4) In 50% of the intervention practices, the GP had access to the sum scores from their patients’ responses to the Multimorbidity Questionnaire (MMQ) and was instructed to include this in the consultation. This latter intervention component will be reported in a forthcoming publication.

### Control

The practices in the control arm were introduced to the background and aim of the SOFIA study and given a brief overview of the program elements. The length and content of the course and extended consultation were unknown to the control group. Patients in the control arm received usual care, which included free access to primary health care during standard and out-of-office hours.

### Outcomes

1. A participation rate of at least 20% of the contacted general practices and 60% of the contacted patients was regarded as an indication of a feasible recruitment strategy. The lower threshold for attendance of patients, i.e., the retention rate, was set at 90% [9].
2. Fidelity was evaluated from process data registered by the GP in REDCap (Research Electronic Data Capture) [11, 12]. Fidelity criteria were set by the research team. A high level of fidelity was considered achieved when the extended consultation contained a physical examination, a medication review, and a shared plan for future contact and treatment.
3. The MultiMorbidity Questionnaire (MMQ) was developed to provide a measure of needs-based quality of life among patients with multimorbidity [13]. EQ-5D-5L was developed by the EuroQol Group to provide a simple, generic measure of health for clinical and economic assessment [14]. All patients were asked to complete the two outcome questionnaires, the MMQ and the EQ-5D-5 L, before randomization (at baseline, i.e. March 2021), and at the end of the trial (October 2021). We set a lower threshold of 90% for the response rate of both questionnaires.
4. To assess whether the coordinated care program might be able to produce a change in outcomes in a larger-scale trial, elements of the program theory were assessed by exploring whether changes in medications and/or detection of new diseases occurred in the intervention group. Changes in symptoms, diagnoses, and medication prescriptions were assessed and registered during the extended consultations and at follow-up consultations by the GP to obtain a preliminary indication of the effectiveness of the intervention in terms of changes in health care provision. The GP registered in text online the findings from the consultation. As such, it was the GP’s perception that guided what information was registered. This information was only collected in the intervention arm.

### Allocation, concealment, and blinding

The study was conducted as a cluster-randomized trial where all patients connected to a specific general practice were either allocated to the intervention or control group. Practices were randomly assigned on a 2:1 basis using a computer algorithm. Randomisation was disclosed after all general practices were assigned and patient recruitment was completed. Due to the nature of the intervention, it was impossible to blind the participants or other members of the research team.

### Data collection and analysis

Study data were collected and managed using REDCap (Research Electronic Data Capture) [11, 12] hosted at the Open Patient data Explorative Network (OPEN), University of Southern Denmark. REDCap is a secure, web-based software platform designed to support data capture for research studies. The data were analysed in SAS version 9.4 for descriptive purposes using chi-squared tests to investigate statistical differences between allocation groups.

## Results

### Baseline characteristics

Three practices in the intervention group were solo practices compared to none in the control group. The mean number of patients per GP was lower in the intervention group (n = 1582) compared to the control group (n = 1697).

Patient baseline characteristics are shown in Table 1. 64 patients were included in the intervention group and 23 patients were included in the control group. There were no significant differences in baseline characteristics between the intervention and control groups. Included patients had a mean age of 49 years (standard deviation (SD): 15.6), most patients were female, and had a diagnosis of severe depression. The most prevalent medical conditions were other mental illnesses, followed by a BMI above 25, hypertension, musculoskeletal disease, and chronic obstructive pulmonary disease/asthma. Patients in the intervention group tended to have fewer GP contacts in the year preceding the study.

**Table 1:** Baseline characteristics of included patients

		Intervention (n = 64)	Control (n = 23)	p-value*
*Inclusion diagnosis*				
	Severe depression	29 (45.3)	10 (43.5)	
	Bipolar disorder	18 (28.1)	8 (34.8)	0.8
	Schizophrenia	17 (26.6)	5 (21.7)	
*Gender*				
	Female	41 (64.1)	18 (78.3)	0.2
	Male	23 (35.9)	5 (21.7)	
*Age*				
	18–24	3 (4.7)	1 (4.4)	
	25–34	14 (21.8)	4 (17.4)	
	35–44	5 (7.8)	5 (21.7)	
	45–54	16 (25.0)	5 (21.7)	0.7
	55–64	12 (18.8)	4 (17.4)	
	≥ 65	14 (21.9)	4 (17.4)	
*Region of residence*				
	Capital	36 (56.3)	13 (56.5)	1.0
	Zealand	28 (43.8)	10 (43.5)	
*Other medical conditions*				
	Other mental illness	28 (43.8)	6 (26.1)	0.1
	Substance use disorder	7 (10.9)	3 (13)	0.8
	Cardiovascular disease	9 (14.1)	2 (8.7)	0.5
	Hypertension	13 (20.3)	6 (26.1)	0.6
	Diabetes	7 (10.9)	1 (4.4)	0.3
	BMI > 25	17 (26.6)	5 (21.7)	0.6
	Smoking	7 (10.9)	2 (8.7)	0.8
	COPD/ asthma	14 (21.9)	3 (13.0)	0.4
	Metabolic disorder	6 (9.4)	2 (8.7)	0.9
	Musculoskeletal disease	16 (25.0)	5 (21.7)	0.8
	Neurological disease	4 (6.3)	2 (8.7)	0.7
	Cancer	1 (1.6)	1 (4.4)	0.4
	Other	14 (21.9)	0 (0.0)	0.01
*Contacts to the clinic in preceding year*				
	0–5	15 (23.4)	2 (8.7)	
	6–10	17 (26.6)	2 (8.7)	
	11–15	11 (17.2)	6 (26.1)	0.06
	16–20	3 (4.7)	5 (21.7)	
	>20	16 (25.0)	7 (30.4)	
	Missing value	2 (3.1)	1 (4.4)	

COPD: Chronic Obstructive Pulmonary Disease

Summary statistics are shown in number and column percentages between parentheses

*p-value based on a 0.05 significance level (Fischer’s Exact Test)

### Recruitment and retention

**Fig. 1:**
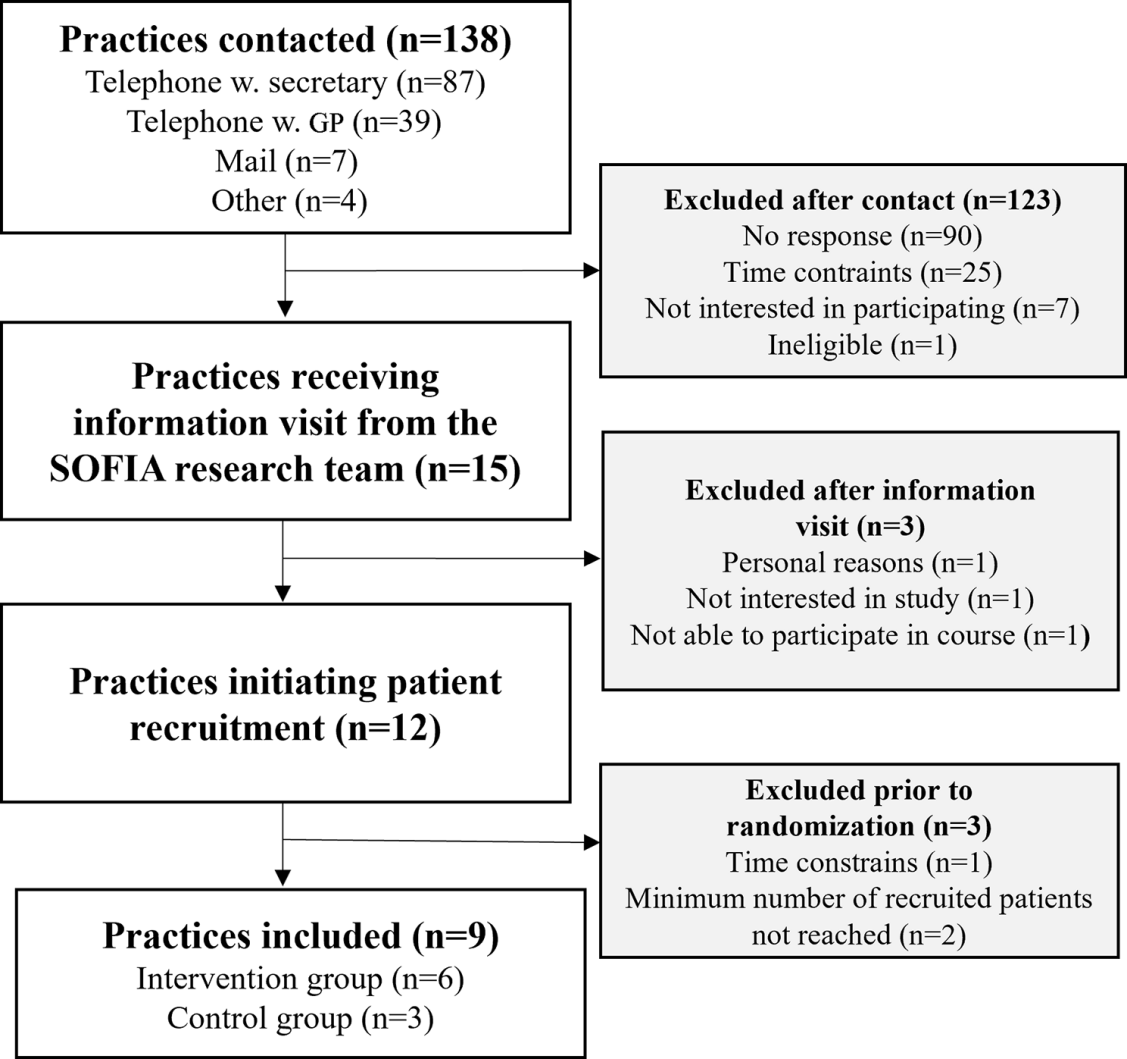
Flowchart visualizing the practice recruitment process in the SOFIA pilot study

Figure 1 visualizes the process of practice recruitment. Of the 138 practices contacted by the research team, 12 practices were included in the study (8.7%). As such the 20% threshold for practice participation was not reached. The main reason for practices not participating was non-response despite multiple contact attempts. Three practices were excluded from the study before randomization due to insufficient patient recruitment (< six patients per practice), amounting to a practice retention rate of 75%.

**Fig. 2:**
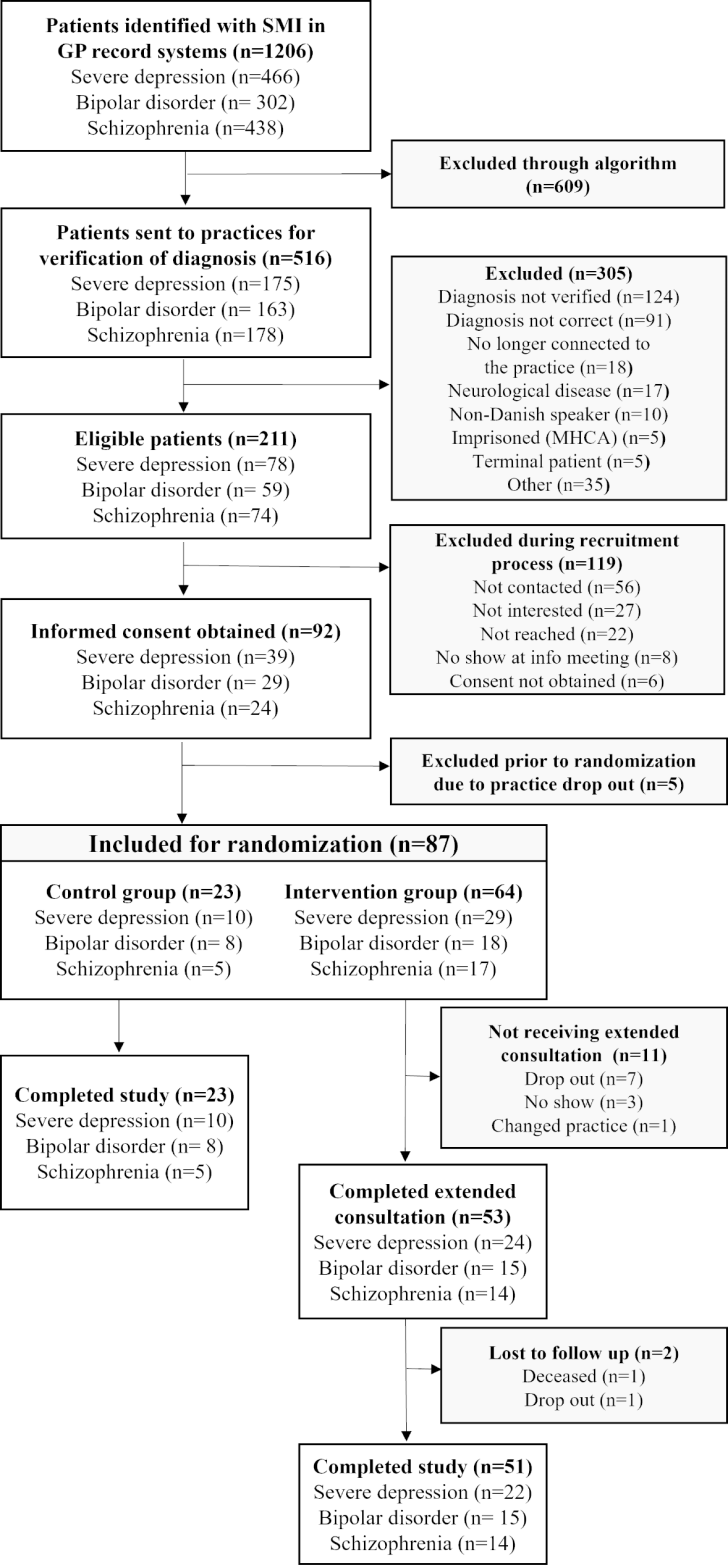
Flowchart visualizing the patient recruitment process in the SOFIA pilot study

Figure 2 depicts the patient recruitment process. Of the 516 patients selected by the algorithm for verification of diagnosis, 305 patients were excluded mostly because the diagnosis was not verified (n = 124 (40.7%)) or because the diagnosis was found to be incorrect by the GP (n = 91 (21%)). 35 patients were excluded due to “other reasons”, without the GP having contacted the research team as instructed. In total, 87 patients were included, with a range of 6–18 patients per practice. 74 patients completed the study resulting in a retention rate of 85.1%. In the intervention group, one patient died for reasons unrelated to the intervention, and one patient dropped out. In the control group, no patients actively dropped out of the study. The patient participation rate (percentage of all patients who signed the informed consent n = 92 out of all patients contacted n = 155) was 59.4% (n = 92) - slightly lower than the desired rate of 60%. Of the 64 patients in the intervention arm, 53 patients (82.8%) attended an extended SOFIA consultation with their GP, slightly below the pre-set attendance rate of 90%.

### Fidelity of the program delivery

The average time of the consultation was 45 min (SD 10.1), and the mean preparation time was 10 min (SD 4.5). 43 of the 53 patients (81.1%) received a physical examination, 50 patients (94.3%) received a medication review, for 49 patients (92.5%) a treatment plan was made, and 40 patients (69.6%) had a follow-up appointment scheduled.

### Feasibility of questionnaire collection

Response rates are shown in Table 2. Patients in the intervention group had the highest baseline response rate at 87.5%. The predetermined response rate threshold of 90% was reached for neither the intervention nor the control group. Response rates in both groups declined to around 70% by the end of the study. No significant differences between the intervention and control groups were detected regarding partially- and fully completed questionnaires.

**Table 2:** Response rates of partially- and fully completed questionnaires

		Baseline		End of Study	
		Initiated	Completed	Initiated	Completed
MMQ1					
	Intervention	56 (87.5%)	56 (87.5%)	44 (68.8%)	44 (68.8%)
	Control	19 (82.6%)	18 (78.3%)	17 (73.9%)	16 (69.5%)
MMQ2					
	Intervention	56 (87.5%)	54 (84.4%)	43 (67.2%)	43 (67.2%)
	Control	18 (78.3%)	18 (78.3%)	16 (69.5%)	16 (69.5%)
EQ-5D-5 L					
	Intervention	56 (87.5%)	56 (87.5%)	44 (68.8%)	44 (68.8%)
	Control	19 (82.6%)	19 (82.6%)	16 (69.5%)	16 (69.5%)

NB: Numerator in response rate calculations is based on number of patients enrolled at baseline

(n = 64 in the intervention group and n = 23 in the control group)

### Changes in symptoms, diagnoses, and medication prescriptions

An overview of new symptoms and diseases detected during the extended consultations is provided in Table 3. In total, new symptoms or diseases were found in 24 patients in the intervention group (47.1%). At the end of follow-up, this number had increased to 31 patients (55.3%). Changes in medication prescription were conducted in 21 (37.5%) of the extended consultations. At the end of the follow-up, the GPs reported changes in medication prescriptions in 29 patients (51.2%) in the intervention group.

**Table 3:** Overview of new diagnoses or symptoms detected during extended- and follow-up consultations

Diagnosis/symptoms	Cases (n)
Dermatological symptoms	5
Hypertension	4
Exacerbation of psychiatric symptoms	4
Shoulder pain (unspecified)	4
Gastrointestinal symptoms	3
Back pain	3
Sleeping disorder	3
Urinary tract symptoms	3
Pelvic pain	2
Weight gain	2
Breast pathology	1
Fibromyalgia	1
Headache	1
Hypermobility of the joints	1
Knee pain	1
Chronic Obstructive Pulmonary Disease	1
Lipoma	1
Medication side effects	1
Metabolic disorder	1
Dizziness	1
Otitis	1

## Discussion

### Statement of principal findings

In this pilot study, we evaluated the fidelity and feasibility of recruitment, program delivery and data collection of a general practice-based coordinated care program targeting patients with severe mental illness (SMI). Our findings indicate that it is feasible for GPs to deliver an extended consultation with relatively high fidelity to the quantifiable components of the SOFIA scheme. Furthermore, the findings suggest that the SOFIA program might assist the GPs in finding new or undetected symptoms and diseases in the patients which supports the underlying program theory. However, the assessment of the recruitment of general practices and patients with SMI for the pilot study revealed challenges in reaching the predetermined participation and retention rates. Moreover, we were not successful in reaching our predetermined response rate threshold of 90% when it came to questionnaire data collection. Although, missing data and incomplete responses did not differ markedly between the intervention and control groups.

### Findings in relation to research and practice

Education of primary care staff, extended consultations for vulnerable patient groups, and various support schemes for health care providers [15–18] are components similar to the SOFIA coordinated care model that have been investigated in other studies and likewise have been shown to be feasible to implement in a general practice setting. However, these components have not been combined in a coordinated model of care, nor have they been assessed among patients with SMI. Thus, this study adds insight into implementing a coordinated care program targeting patients with SMI. Before the SOFIA pilot study, the SOFIA scheme (which is limited to the extended consultation) was tested in a small feasibility study [10]. In the smaller-scale feasibility study, GPs and patients found the extended consultation acceptable, however, the consultations were delivered with low fidelity. In contrast, this pilot study found that extended consultations were delivered with relatively high fidelity to the SOFIA scheme, illustrating the value of the synergistic effects of the entire coordinated care program. Further, the tentative findings add to the potential effects of extended consultations in changing patients’ care plans by providing quantitative evidence in support of altered diagnoses and changes in the patients’ medication prescriptions [19]. Moreover, the cluster randomisation showed no differences in baseline characteristics between allocation groups which shows us that the randomisation setup proved successful. In an international context, the CARE Plus study is comparable to the SOFIA study, which aimed to enhance primary health care for patients from severely deprived areas in Glasgow [15]. The CARE Plus study demonstrated that a structured extended consultation in general practice was feasible and somewhat effective to implement for patients with multimorbidity. In our study, we add to this finding, by highlighting that a coordinated care program can likewise be implemented with high fidelity to patients with SMI in general practice [15].

After the completion of the SOFIA pilot study, elements of the extended consultation were included in a national general practice agreement in Denmark in 2022. Hence, it is now possible for GPs to receive reimbursement for yearly extended consultations for patients with SMI focused on the detection and treatment of somatic diseases. The consultation in the agreement resembles the structure of the extended SOFIA consultation, but the agreement does not include an educational course nor a specialized handbook of relevant municipal and regional support systems. With extended consultations now being possible nationwide, a future SOFIA RCT is not applicable. However, the findings of this study will inspire the implementation of the consultations in the new agreement.

### Strengths and weaknesses

The primary strength of the SOFIA study lies in the thorough theoretical and methodological foundation that formed the development of the coordinated care program. Inspired by the Medical Research Council (MRC) guidelines, a comprehensive co-design phase involving a variety of stakeholders, collection and reviewing of existing evidence in combination with anthropological fieldwork, informed the development of the coordinated care program and the underlying program theory [8].

The fidelity assessment of the extended consultations was based on GP-reported outcomes, which may have led to an overestimation of the performed fidelity in the study. It can be argued that continuous registration of fidelity measures while delivering the intervention serves as a constant reminder to the GPs. This could mean that REDCap registration represents an active intervention element, which might have resulted in elevated fidelity in the study. Lastly, our data only provided insight regarding the extent to which the activities were conducted, but not the quality of the implementation.

Recruitment of practices appeared to be a substantial challenge in our study. Other interventional studies conducted in general practice settings have reported recruitment rates varying from 2 to 81% [20]. The most common reasons for declining participation are either time constraints or non-response [20], which accords with the findings in our study. In the SOFIA study, the practice recruitment rates were low despite using multiple concurrent strategies as recommended in the literature to heighten recruitment success [21]. These strategies entailed mail and telephone invitations, verbal presentations, workshops, and announcements in newsletters. The COVID-19 pandemic might have contributed considerably to the perceived time constraint in the contacted general practices, and therefore discouraged study participation but most likely it does not in itself explain this lack of recruitment success. The low recruitment rate of practices makes it relevant to consider volunteer “bias”, i.e. selection of practices that are not representative of the general practices in Denmark. It is likely, that practices willing to participate in a study targeting patients with SMI, already have a particular focus or interest in this patient group. This means that we might not have fully assessed the implementation fidelity and feasibility of the SOFIA study if recruited practices differ from non-recruited practices.

Similarly, we could not fully eliminate the risk of selective patient recruitment. Despite relatively strict in- and exclusion criteria, a large proportion of patients were excluded due to “other reasons”, suggesting that GPs, to a certain extent, selectively enrolled participants. Numerous electronic medical record systems are used in Danish general practices, which hampers a single, standardized approach to identifying eligible patients, and the accuracy and degree of completeness of patient diagnoses in the medical records are known to present substantial variation [22]. The reported patient retention rate was likewise not reached and an examination of the benefits and potential disadvantages of the recruitment strategy and GPs’ adherence to recruitment strategy will be explored in-depth qualitatively in a future publication. In addition, patient recruitment challenges also explained the relatively low retention rate of general practices (75%).

New diagnoses, symptoms, and medication prescriptions were registered in approximately half of the patients in the intervention group, suggesting that the coordinated care program may bring about changes in healthcare provision. However, no new cases of cardiovascular disorders or diabetes mellitus were detected - the conditions that confer the greatest risk of premature death in this patient group [5]. Moreover, data regarding the detection of new symptoms and diseases was not collected in the control group meaning that an assessment of the efficacy and effectiveness is not possible in this study. The actual effectiveness of the intervention in reducing mortality needs to be evaluated in the newly implemented GP agreement.

### Implications for future research

In conclusion, our results demonstrate that it is feasible to deliver a coordinated care program consisting of extended structured consultations carried out by the GP, group-based training of GPs and staff, and signposting of patients to relevant municipal, health, and social initiatives in a cluster-randomized controlled setting. However, the recruitment strategy concerning practices and patients should be revised before proceeding to evaluate the effectiveness of the intervention. Successful implementation may open new avenues for the development of evidence-based methods to reduce inequalities or even inequities, in the provision of primary health care in patients with SMI.

## Data Availability

The datasets analysed during the current study are not publicly available since they contain individual information on patients and practitioners. For inquiries on data requests please contact the corresponding author K. Tranberg (katrine.tranberg.jensen@sund.ku.dk).

## Abbreviations

GP/GPs: General practitioner/s
MMQ: Multimorbidity Questionnaire
SMI: Severe mental illness
SOFIA: SOcial, FInding, and Agreement

## References

[1] Hjorthoj C, Sturup AE, McGrath JJ (2017). Years of potential life lost and life expectancy in schizophrenia: a systematic review and meta-analysis. Lancet Psychiatry.

[2] Chang CK, Hayes RD, Perera G (2011). Life expectancy at birth for people with serious mental illness and other major disorders from a secondary mental health care case register in London. PLoS ONE.

[3] Oude Voshaar RC, Aprahamian I, Borges MK (2021). Excess mortality in depressive and anxiety disorders: the Lifelines Cohort Study. Eur Psychiatry.

[4] Lawrence D, Hancock KJ, Kisely S (2013). The gap in life expectancy from preventable physical illness in psychiatric patients in western Australia: retrospective analysis of population based registers. BMJ.

[5] Laursen TM, Munk-Olsen T, Gasse C (2011). Chronic somatic comorbidity and excess mortality due to natural causes in persons with schizophrenia or bipolar affective disorder. PLoS ONE.

[6] Henderson DC, Vincenzi B, Andrea NV (2015). Pathophysiological mechanisms of increased cardiometabolic risk in people with schizophrenia and other severe mental illnesses. Lancet Psychiatry.

[7] Baxter AJ, Harris MG, Khatib Y (2016). Reducing excess mortality due to chronic disease in people with severe mental illness: meta-review of health interventions. Br J Psychiatry.

[8] Reventlow SJA, Møller MCR, Davidsen AS, Olsen L. Co-producing Healthcare Interventions: transforming Transdisciplinary Research to develop Healthcare Services to meet the needs of patients with complex problems. In: Sturmberg J, editor. Embracing complexity in Health: the Transformation of Science, Practice, and policy. Volume 1. ed: Springer International Publishing; 2019.

[9] Rozing MP, Jonsson A, Koster-Rasmussen R (2021). The SOFIA pilot trial: a cluster-randomized trial of coordinated, co-produced care to reduce mortality and improve quality of life in people with severe mental illness in the general practice setting. Pilot Feasibility Stud.

[10] Jønsson ABR, Martiny FHM, Søndergaard MK et al. Introducing Extended Consultations for Patients with Severe Mental Illness in General Practice. Research Unit for General Practice Copenhagen, Section for General Practice, University of Copenhagen. 10.21203/rs.3.rs-1983933/v1.

[11] Harris PA, Taylor R, Minor BL (2019). The REDCap consortium: building an international community of software platform partners. J Biomed Inform.

[12] Harris PA, Taylor R, Thielke R (2009). Research electronic data capture (REDCap)--a metadata-driven methodology and workflow process for providing translational research informatics support. J Biomed Inform.

[13] Bissenbakker KHMA, Brodersen J, Jønsson ABR. Conceptualisation of a Measurement Framework for needs-based quality of life among patients with multimorbidity [Manuscript submitted for publication]. Research Unit for General Practice Copenhagen, Section for General Practice, University of Copenhagen; 2022.

[14] Herdman M, Gudex C, Lloyd A (2011). Development and preliminary testing of the new five-level version of EQ-5D (EQ-5D-5L). Qual Life Res.

[15] Mercer SW, Fitzpatrick B, Guthrie B (2016). The CARE Plus study - a whole-system intervention to improve quality of life of primary care patients with multimorbidity in areas of high socioeconomic deprivation: exploratory cluster randomised controlled trial and cost-utility analysis. BMC Med.

[16] Larsen JR, Siersma VD, Davidsen AS et al. The excess mortality of patients with diabetes and concurrent psychiatric illness is markedly reduced by structured personal diabetes care: a 19-year follow up of the randomized controlled study Diabetes Care in General Practice (DCGP). Gen Hosp Psychiatry 2016 Jan-Feb;38:42–52. 10.1016/j.genhosppsych.2015.10.001. PubMed PMID: 26602087. 10.1016/j.genhosppsych.2015.10.001 26602087

[17] Pereira Gray DJ, Sidaway-Lee K, White E (2018). Continuity of care with doctors—a matter of life and death? A systematic review of continuity of care and mortality. BMJ Open.

[18] Smith SM, Wallace E, O’Dowd T, et al. Interventions for improving outcomes in patients with multimorbidity in primary care and community settings. Cochrane Database of Systematic Reviews. 2016;310.1002/14651858.CD006560.pub3. PubMed PMID: CD006560. 10.1002/14651858.CD006560.pub3 PMC6703144 26976529

[19] Bonfils IS. Implementing the Individual Placement and Support approach in institutional settings for employment and mental health services – perceptions and challenges from a case study in Denmark. Eur J Social Work. 2021;1–14. 10.1080/13691457.2020.1870216.

[20] Sahin DYM, Sussman T, McCusker J. A mixed studies literature review of family physicians’ participation in research. Fam Med. 2014 Jul-Aug;46(7):503–14. PubMed Central PMCID: PMC25058542. 25058542

[21] Tan ACW, Clemson L, Mackenzie L (2019). Strategies for recruitment in general practice settings: the iSOLVE fall prevention pragmatic cluster randomised controlled trial. BMC Med Res Methodol.

[22] Schroll HS, Kragstrup J (2003). Forskelle i praktiserende lægers anvendelse af International classification for primary Care-diagnoser. Inter- og intraobservatørvariationen. Ugeskrift for Læger.

